# Editorial: Inflammation and blood diseases, a dog chasing its tail

**DOI:** 10.3389/fonc.2023.1180559

**Published:** 2023-03-24

**Authors:** Cosimo Cumbo, Francesco Tarantini, Michele Gottardi, Francesco Albano

**Affiliations:** ^1^ Department of Precision and Regenerative Medicine and Ionian Area (DiMePRe-J), Hematology and Stem Cell Transplantation Unit, University of Bari “Aldo Moro”, Bari, Italy; ^2^ Onco Hematology, Veneto Institute of Oncology IOV, IRCCS, Padua, Italy

**Keywords:** inflammation, ENKTL, MDS, IBD, HLH

The close link between inflammation and hematological malignancies has been widely shown (1). It is known how inflammation regulates the hematopoietic stem cell (HSC) fate, affecting its proliferation, differentiation and self-renewal abilities (1). On the other hand, the blood disease itself or specific therapeutic approaches can elicit an inflammatory response, thus playing a major role in disease progression and establishing a vicious circle that is yet to be explored. Furthermore, in the last years, a growing interest is emerging in the possible interplay between chronic inflammation, aging and hematological malignancies (1).

Inflammation can be the fertile ground in which blood diseases may arise. In a recent retrospective analysis, Lei et al. investigated the effect of chronic rhinosinusitis (CRS) on the staging and prognosis of extranodal natural killer/T-cell lymphoma (ENKTL). The authors demonstrated that the length of CRS history was correlated with the stage of ENKTL, whose primary sites were the upper aerodigestive tract (UAT). CRS history was an independent prognostic predictor for progression-free survival of the UAT-ENKTL patients.

The role of chronic inflammatory stimulus as a prognostic predictor was also explored by Shi et al. in the context of myelodysplastic syndromes (MDS). The authors examined the predictive value of certain pretherapy peripheral blood inflammatory indices: platelet-to-lymphocyte ratio (PLR), neutrophil-to-lymphocyte ratio (NLR), and plasma C-reactive protein (CRP). Notably, elevated PLR and CRP predict poor prognosis independent of the Revised International Prognostic Scoring System (IPSS-R) score and provide a novel evaluation factor for MDS patients.

The contribution of new inflammatory indices in the outcome of blood diseases has been recently studied also in the context of myeloproliferative neoplasms (MPNs), nowadays considered a “Human Inflammation Model”; the disease itself produces a state of chronic inflammation due to the continuous release of inflammatory molecules from activated leukocytes and platelets (2, 3). Increasing release of cytokines, chemokines, and reactive oxygen species (ROS) production gives rise to genetic and epigenetic changes, inducing genomic instability and thereby contributing to tumor fitness (4). All these considered, the role of interferon regulatory factor 4 (*IRF4*), acting in several immunosurveillance and inflammatory mechanisms (i.e.: regulation of myeloid-derived suppressor cells (MDSCs) and polarization of anti-inflammatory M2 macrophages) was recently investigated in MPNs. *IRF4* is downregulated in these disorders, and its expression is associated with the disease outcome (5, 6).

The CRS history in the outcome of the UAT-ENKTL patients, the elevated PLR and CRP in the context of MDS and the *IRF4* expression in MPNs can be considered new examples of “inflammatory parameters”, impacting the prognosis of three different hematological diseases (Lei et al.; Shi et al., 5, 6).

In some circumstances, the relationship between inflammation and the disease onset is clearly described, and each player takes a specific role; in other circumstances, it is not clear if the first causes the latter or vice-versa. Several cellular and molecular mechanisms are involved in this interplay; among them, in the last years, many studies focused on clonal hematopoiesis (CH) onset in multiple physio-pathological (even hematological) conditions (7–10). A growing body of evidence suggests a correlation between inflammation and CH. On the one hand, an inflammatory state may favor the selection and emergence of a mutated hematopoietic clone; on the other hand, the mutated clone itself may increase the expression of inflammatory genes in innate immune cells (7). Recently, our group suggested that a CH event could represent a possible link between inflammatory bowel diseases (IBDs) and blood cancer onset (Cumbo et al.). As demonstrated, an IBD may promote the CH event (11); the clone selected could evolve in a subsequent blood disease or promote its insurgence. In both cases, the inflammatory milieu of the hematological malignancy may, in turn, feed the IBD (Cumbo et al.). The detection of a CH event in a patient carrying a chronic inflammatory disease such as IBD should require a hematologic follow-up to assess the risk of developing a blood cancer (Cumbo et al.). Future studies will clarify the relationship between these conditions.

In the intricate interaction between inflammation and hematopoiesis, rheumatologic diseases need to be considered. Ren et al. describe the onset in pregnancy of a rare and severe clinical syndrome: the hemophagocytic lymphohistiocytosis (HLH), in the “fertile ground” of systemic lupus erythematosus (SLE). Apart from this rare combination of events, it is known that SLE patients have an increased risk of developing a hematological malignancy than the general population, especially for those at a higher age when diagnosed with SLE (12). Furthermore, the prevalence of CH is high in SLE after controlling for age, and CH occurs >20 years earlier in SLE than in controls (13). Another example of the possible crosstalk between systemic inflammation, CH events and blood cancer onset? The issue has not gone away.

Furthermore, it is conceivable that, in some circumstances, inflammation might be considered an opportunity to exploit, as demonstrated in refractory acute myeloid leukemia (AML) (14). A recent multicenter study showed the efficacy of flotetuzumab (a bispecific antibody-based molecule to CD3 and CD123) as salvage immunotherapy in these patients. The authors showed the association between an immune-infiltrated tumor microenvironment and resistance to cytarabine-based chemotherapy but responsiveness to flotetuzumab (14).

Between several new or well-known mechanisms (disease-microenvironment crosstalk, immune cells activity and cytokines production, onset and effects of CH, the role of chronic inflammation, new inflammatory indicators and more genetic markers description), a particular interest is now addressed to new data impacting on disease outcome and opening new scenarios for clinical practice and diseases’ management. Molecular and/or cellular inflammatory parameters could be considered new prognostic markers to take into account in different blood diseases. The CH detection in the background of inflammatory disease could suggest a hematologic follow-up for the patient; last but not least, the CH onset during the follow-up of a hematological patient may impact its long-term clinical management, as recently shown in chronic myeloid leukemia (CML) (9). In these patients, as in other contexts of clinical practice, the occurrence of CH could reveal a novel player that needs to be taken into account (8).

All recent experimental and clinical findings paint a fascinating but incomplete picture of the complex interaction between inflammation and blood diseases, two active players placed in a widely studied vicious circle, offering intriguing open questions.

## Author contributions

Conception and design of the study: CC and FA. Manuscript preparation: CC, FT, MG and FA. Drafting of the text: FA. All authors revised the manuscript for important intellectual content and approved the final version submitted for publication.
